# Development of *CypriSSR*: a genome-wide, chromosome-level microsatellite database for multiple cyprinidae species

**DOI:** 10.1093/database/baag035

**Published:** 2026-06-29

**Authors:** Princy Saini, Mir Asif Iquebal, Sarika Jaiswal, Ulavappa B Angadi

**Affiliations:** Division of Agricultural Bioinformatics, ICAR-Indian Agricultural Statistics Research Institute, Library Avenue, PUSA, New Delhi 110012, India; The Graduate School, ICAR-Indian Agricultural Research Institute, Library Avenue, PUSA, New Delhi 110012, India; Division of Agricultural Bioinformatics, ICAR-Indian Agricultural Statistics Research Institute, Library Avenue, PUSA, New Delhi 110012, India; Division of Agricultural Bioinformatics, ICAR-Indian Agricultural Statistics Research Institute, Library Avenue, PUSA, New Delhi 110012, India; Division of Agricultural Bioinformatics, ICAR-Indian Agricultural Statistics Research Institute, Library Avenue, PUSA, New Delhi 110012, India

## Abstract

The family *Cyprinidae* represents the most taxonomically diverse group of freshwater fishes, encompassing over 3 000 species of ecological and economic importance in aquaculture, conservation, and ecological monitoring. Simple sequence repeats (SSRs), also known as microsatellites, are highly informative molecular markers widely used for genetic diversity analysis, population structure assessment, and marker-assisted breeding. However, comprehensive genome-wide SSR resources for cyprinids remain limited. Existing databases, such as FishMicrosat, provide restricted taxonomic coverage and lack standardized chromosome-level datasets suitable for comparative genomic analyses. To address this gap, we developed *CypriSSR*, a genome-wide SSR database encompassing 11 representative cyprinid species. Chromosome-level genome assemblies were retrieved from NCBI. SSR loci were identified using MISA, and primer pairs were designed using Primer3. In total, over 7.8 million SSR loci were identified. Mononucleotide repeats were the most abundant class (39–53%), followed by dinucleotide and trinucleotide motifs. SSRs were predominantly distributed in genic regions (54%–71% across species), suggesting potential functional roles. Each database entry includes repeat type, genomic coordinates, primer sequences, melting temperatures, and predicted PCR product sizes. The *CypriSSR* web interface enables flexible querying of SSR markers based on species, chromosome, motif class, and genomic location, and supports sequence similarity searches through an integrated BLAST module along with data export options. *CypriSSR* provides a comprehensive and standardized multi-species microsatellite resource for cyprinid genomes and supports applications in population genetics, molecular breeding, and conservation genomics. **Database URL:**  http://46.202.167.198/fishssr/

## Introduction

Global freshwater ecosystems are under unprecedented pressure from habitat destruction, pollution, climate change, and overexploitation, leading to accelerated biodiversity loss and threatening the sustainability of inland fisheries and aquaculture [1–3]. Land-use change, pollution, habitat fragmentation, invasive species, and climate change have collectively intensified biodiversity loss in freshwater systems at rates exceeding those observed in terrestrial and marine ecosystems [4,5]. The Intergovernmental Science-Policy Platform on Biodiversity and Ecosystem Services (IPBES) has estimated that freshwater vertebrate populations have declined by approximately 83% since 1970, highlighting the urgent need for enhanced biodiversity monitoring, sustainable resource management, and effective conservation strategies [6,7].

Within these ecosystems, the family Cyprinidae represents the most species-rich lineage of freshwater fishes, comprising over 3 000 species distributed across Eurasia, Africa, and North America [8]. Cyprinids occupy a wide range of ecological niches and function as keystone species, ecological indicators, and ecosystem engineers. They also dominate global inland aquaculture production according to the Food and Agriculture Organization (FAO) [9]; species such as *Cyprinus carpio* (common carp), *Labeo rohita* (rohu), *Ctenopharyngodon idella* (grass carp), and *Hypophthalmichthys molitrix* (silver carp) collectively contributed over 32 million tonnes to global aquaculture production in 2022, playing a critical role in food security, particularly across Asia. Beyond aquaculture, the family encompasses invasive taxa such as *H. molitrix, Hypophthalmichthys nobilis*, and *Mylopharyngodon piceus*, which disrupt native ecosystems, as well as high-value ornamental species including *Carassius auratus* (goldfish) and *Puntigrus tetrazona* (tiger barb). Additionally, species such as *Squalius cephalus* (chub) and *Alburnus alburnus* (bleak) are widely used as bioindicators in ecological monitoring programs. This ecological, economic, and biogeographical diversity underscores the importance of conserving and characterizing cyprinid genetic resources [10–12].

Genetic variation underlies population resilience to environmental stressors, disease outbreaks, and climate change. In aquaculture systems, maintaining genetic diversity is essential for the development of robust and adaptable stocks, enhancing yield, and minimizing the risks associated with inbreeding depression [13,14]. From a conservation perspective, genetic data inform the delineation of evolutionarily significant units, support ex situ breeding and reintroduction programs, and enable monitoring of invasive or hybrid populations [15]. Understanding cyprinid genomic architecture thus supports both biodiversity conservation and the sustainable management of fisheries and aquaculture resources [14].

Microsatellites, also referred to as simple sequence repeats (SSRs), are tandemly repeated DNA sequences composed of mono-, di-, tri-, tetra-, penta-, or hexa-nucleotide motifs that occur abundantly and ubiquitously across eukaryotic genomes. Owing to their high polymorphism, broad genomic distribution, and codominant mode of inheritance, SSRs rank among the most powerful molecular markers for population genetic investigations [16–19]. Their robust polymorphism makes them ideal for studies of genetic linkage maps [20–22], population genetics [23], diversity assessment, molecular breeding, and allele mining [24,25]. In cyprinid species, which exhibit considerable genomic variability across wild, cultured, and invasive populations, SSRs provide a robust framework for resolving population structure, monitoring temporal genetic dynamics, and supporting selective breeding programs aimed at sustainable aquaculture [26–28].

Despite the established utility of SSRs, currently available resources for cyprinid and related fish species remain limited in both scale and methodological consistency. Existing databases, such as FishMicrosat database [29], have primarily been developed using microsatellite sequences derived from public repositories and encompass a relatively limited number of species and loci. However, their reliance on sequence-derived datasets rather than systematic genome-wide mining restricts the completeness and resolution of SSR markers. Moreover, the lack of chromosome-level genomic context, standardized pipelines for SSR identification, and uniform annotation frameworks limits their applicability for large-scale comparative genomic analyses. In addition, restricted taxonomic representation results in the underrepresentation of several ecologically and economically important cyprinid species. Collectively, these limitations constrain the effective application of SSR markers in population genetics, molecular breeding, and conservation genomics within the Cyprinidae family. Therefore, there is a clear need for a unified, genome-wide, multi-species SSR resource that integrates systematic SSR discovery from publicly available genome assemblies with standardized annotation and chromosome-level resolution.

To address these limitations, we developed *CypriSSR* (http://46.202.167.198/fishssr/), a genome-wide SSR database encompassing 11 representative cyprinid species. SSR loci were identified from chromosome-level genome assemblies using MISA, and primers were designed with Primer3. In total, more than 7.8 million SSR loci were identified and annotated with repeat motifs, genomic coordinates, repeat counts, genic and intergenic classification, primer sequences, melting temperatures, and predicted product sizes, along with information on potential cross-species polymorphism. The database provides a user-friendly web interface that supports flexible queries across species, chromosomes, motif types, and genomic regions, and includes an integrated BLAST module along with data download options. *CypriSSR* provides a genome-wide, chromosome-level SSR resource for cyprinid species and supports applications in population genetics, molecular breeding, and conservation genomics.

## Materials and methods

### Data source

Genomic data for 11 representative cyprinid fish species were retrieved from NCBI (www.ncbi.nlm.nih.gov), accessed in September 2025 [30]. The selected species represent diverse ecological and economic relevance, including major aquaculture species (e.g. *C. carpio, L. rohita, C. idella*), invasive species (*H. molitrix, H. nobilis, M. piceus*), ornamental fishes (*C. auratus, P. tetrazona*), and ecological indicator species (*S. cephalus, A. alburnus*).

For each species, chromosome-level genome assemblies were downloaded in FASTA format, along with corresponding gene annotation files (GFF or GTF) when available. A summary of genome assembly characteristics, including assembly accession, assembly name, assembly level, genome size, GC content, and chromosome number, is provided in Table 1. Genome assemblies were obtained from both GenBank (GCA) and RefSeq (GCF) repositories based on availability.

**Table 1.** Summary of genome assemblies used for genome-wide microsatellite identification in cyprinid species.

Species	Assembly accession	Assembly name	Assembly level	Genome size (mb)	Gc %	No. of chromosomes
*Cyprinus carpio*	GCA_018340385.1	ASM1834038v1	Chromosome	1700	37	50
*Labeo rohita*	GCA_022985175.1	IGBB_LRoh.1.0	Chromosome	1100	36	25
*Carassius gibelio*	GCA_023724105.1	carGib1.2-hapl.c	Chromosome	1600	37.5	50
*Carassius auratus*	GCA_003368295.1	ASM336829v1	Chromosome	1800	37.5	59
*Ctenopharyngodon idella*	GCA_019924925.1	HZGC01	Chromosome	893.2	37.5	24
*Hypophthalmichthys molitrix*	GCA_037950675.1	Hypophthalmichthys_molitrix_1.0	Chromosome	853.5	37.5	24
*Hypophthalmichthys nobilis*	GCA_037950665.1	Hypophthalmichthys_nobilis_1.0	Chromosome	863.9	37.5	24
*Mylopharyngodon piceus*	GCA_039654765.1	ASM3965476v1	Chromosome	848.7	37.5	24
*Puntigrus tetrazona*	GCF_018831695.1	ASM1883169v1	Chromosome	730.8	38	25
*Squalius cephalus*	GCA_022829025.1	ASM2282902v1	Chromosome	1000	38.5	25
*Alburnus alburnus*	GCA_964188515.1	fAlbAlb.hap1	Chromosome	1000	38.5	25

### Identification of genome-wide SSRs

Genome-wide simple sequence repeats (SSRs) were identified from the chromosome-level genome assemblies using the Microsatellite Identification Tool (MISA) [31]. SSR detection was performed using minimum repeat thresholds of 10 units for mononucleotide motifs, 6 units for dinucleotide motifs, and 5 units for tri-, tetra-, penta-, and hexanucleotide motifs. These thresholds were chosen following commonly used criteria in genome-wide SSR studies and have been widely applied for consistent identification of SSR loci [32,33]. SSR loci separated by less than 100 base pairs were classified as compound SSRs. For each genome, MISA generated two output files: (i) a detailed locus file containing genomic coordinates, repeat motifs, and repeat counts for individual SSRs, and (ii) a summary file describing the overall abundance and distribution of SSR motifs across the genome.

To functionally annotate SSR loci, the genomic coordinates of identified SSRs were intersected with species-specific gene annotation files (GFF/GTF) using BEDTools [34]. Based on their positional overlap with annotated genomic features, SSRs were categorized into genic regions, including coding sequences (CDS), 5' and 3' untranslated regions (UTRs), and introns, or into non-genic (intergenic) regions. This classification enabled comparative analyses of SSR distribution across distinct functional genomic compartments.

### Primer design for genome-wide SSRs

PCR primers were designed for each identified SSR locus to enable reliable amplification and downstream genotyping. Before primer design, flanking sequences of 500 bp upstream and downstream of each SSR motif were extracted using custom Perl scripts, providing sufficient sequence context while minimizing interference from adjacent repetitive elements. Flanking regions overlapping repeat-masked sequences were excluded to ensure primers were anchored in unique, non-repetitive genomic regions.

Primer design was performed using Primer3 [35]. MISA output files were converted into Primer3-compatible input formats using two Perl scripts *p3_in.pl* and *p3_out.pl*, which automated both input preparation and result parsing. Primers were designed using the following criteria: length of 18–27 bp, melting temperature (Tm) of 57–63°C, GC content of 30–70%, and an expected PCR product size of 100–300 bp. These parameters were selected based on standard guidelines for PCR primer design to ensure specificity, amplification efficiency, and broad applicability across diverse cyprinid genomes [18].

### *In silico* discovery of polymorphic markers

**Figure 1:**
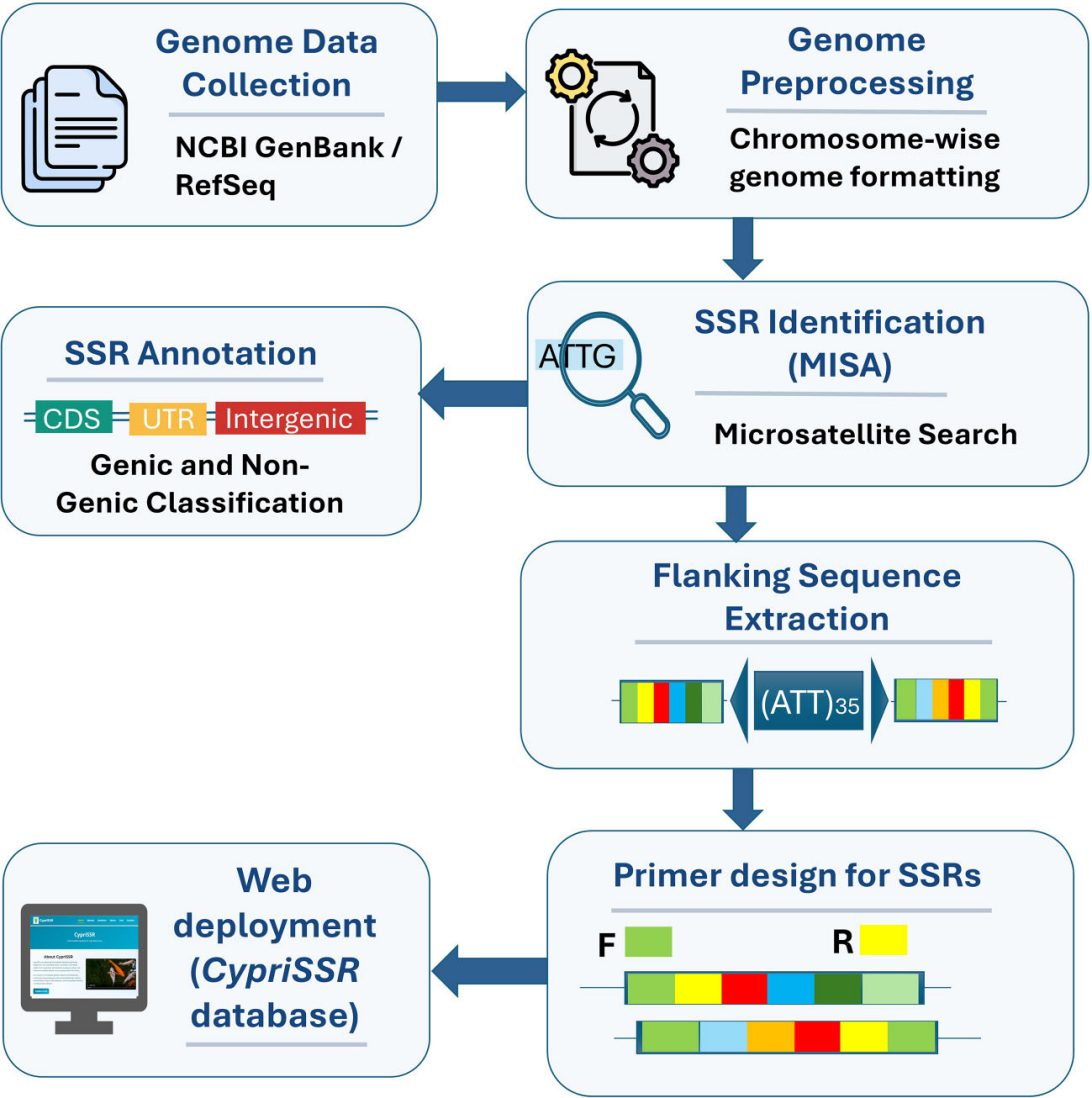
Workflow for genome-wide microsatellite (SSR) identification and development of the *CypriSSR* database.

To enrich for polymorphic markers, SSR loci containing eight or more repeat units were selected prior to primer design, as SSRs with higher repeat numbers are generally associated with increased levels of polymorphism due to elevated replication slippage [36]. Simple SSRs (mono- to hexanucleotide repeats) were retained, whereas compound and complex repeats were excluded due to their reduced amplification reliability. All filtering, flanking sequence extraction, and marker selection steps were automated using in-house Perl scripts. Loci showing variation in predicted PCR product size were considered as potentially polymorphic [37]. The overall workflow for genome-wide SSR identification, primer design, and database integration is illustrated in Fig. 1.

### Development of *CypriSSR* database

**Figure 2:**
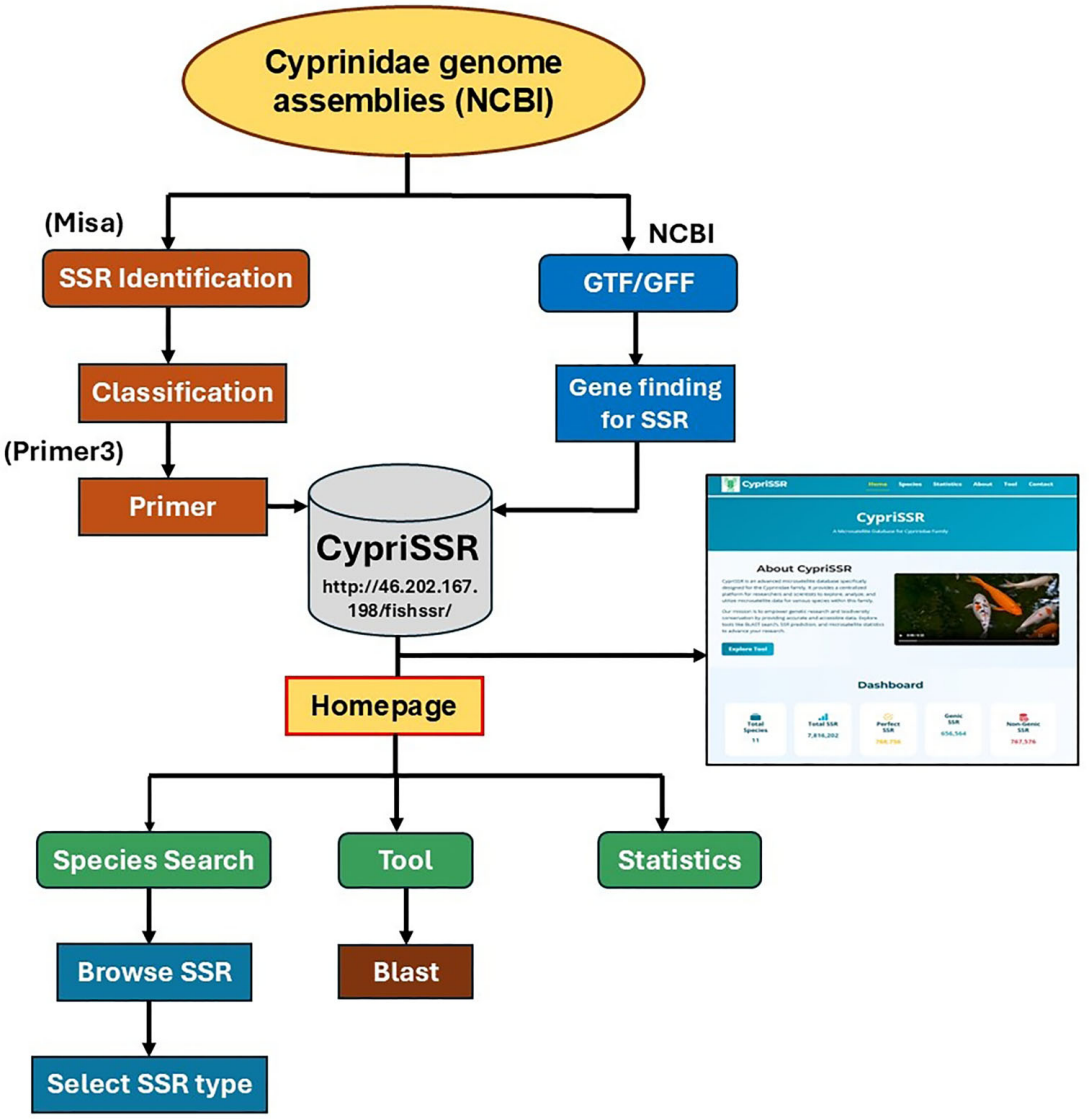
Architecture and workflow of the *CypriSSR* database. Genome assemblies and gene annotation files obtained from NCBI are processed through a computational pipeline for SSR identification, functional classification, and primer design. The resulting data are stored in a MySQL-based backend and accessed through a web-based interface that provides species-specific browsing, SSR filtering, BLAST-based sequence searches, and statistical visualization tools.

*CypriSSR* was developed as a web-based relational database using PHP (version 7.4.3) and MySQL (version 8.0.27) in a standard web development environment. The database architecture was designed based on a three-tier model comprising the presentation layer, application layer, and database layer, ensuring efficient data management, scalability, and user accessibility. At the backend, a MySQL database was constructed to store genome-wide SSR information, including locus details, genomic coordinates, repeat motifs, primer sequences, and genic or non-genic classifications. PHP scripts have been implemented to process user queries, manage data retrieval, and facilitate interaction between the user interface and the database. The presentation layer was developed using HTML, CSS, Bootstrap, and JavaScript to provide an interactive and user-friendly interface. Genome assemblies retrieved from NCBI (Supplementary Table 1) were processed through an integrated pipeline involving SSR identification using MISA, functional classification based on gene annotation files (GFF/GTF), and primer design using Primer3. The resulting datasets were systematically organized and indexed within the *CypriSSR* database to enable efficient querying and retrieval. The overall system architecture and workflow are illustrated in Fig. 2. *CypriSSR* was equipped with multiple utilities for SSR exploration and marker selection. Users can browse SSR markers across species-specific datasets and apply filters based on chromosome, repeat motif type, and genomic location (genic or non-genic). The database also includes modules for visualizing SSR distribution patterns. An integrated BLAST interface was incorporated to support BLASTN and tblastn sequence similarity searches, enabling identification of homologous SSR-associated regions across cyprinid genomes. Additionally, users can download selected data in standard formats such as CSV and Excel for downstream analyses. The database was designed to support researchers in genomics, population genetics, and breeding studies by providing flexible search options and comprehensive SSR marker information. *CypriSSR* was thus established as an accessible and scalable genomic resource for genome-wide SSR analysis and comparative studies in cyprinid species.

## Results and discussion

### Genome-wide distribution and abundance of SSRs

A total of 7,816,202 SSR loci were identified across the genomes of eleven cyprinid species, highlighting the extensive abundance and diversity of SSRs within cyprinid genomes. The overall SSR density ranged from 557.45 to 704.69 SSRs/Mb, with a mean density of approximately 620 SSRs/Mb across species (Supplementary Table 1), indicating substantial variation in repeat content. Among the analyzed genomes, *C. auratus* and *C. carpio* exhibited the highest number of SSRs (1,235,158 and 1,197,973 loci, respectively), consistent with their relatively larger genome sizes. In contrast, *P. tetrazona* and *A. alburnus* showed comparatively lower SSR counts (462,975 and 557,453 loci, respectively). These observations suggest a positive association between genome size and total SSR abundance, consistent with previous reports in eukaryotic and vertebrate genomes [38] (Supplementary Table 1). However, SSR density (SSRs/Mb) did not show a proportional relationship with genome size, indicating that SSR accumulation is not solely dependent on genome expansion. Similar observations have been reported in large-scale comparative analyses, where SSR abundance correlates with genome size, whereas SSR density remains largely independent of genome size [39,40].

In terms of repeat composition, mononucleotide repeats were the most abundant class, accounting for approximately 39–53% of total SSRs across species, followed by dinucleotide repeats, while trinucleotide repeats occurred at moderate frequencies. Tetra-, penta-, and hexanucleotide repeats were relatively rare (Supplementary Table 2). The predominance of shorter repeat motifs is consistent with observations in other teleost genomes and may reflect higher mutation rates associated with replication slippage. A similar decrease in abundance with increasing motif length has been reported in comparative analyses of fish genomes [39].

The density of SSRs per megabase (SSRs/Mb) provides additional insight into genome organization across species. *C. carpio* exhibited the highest SSR density (704.69 SSRs/Mb), followed by *C. auratus* (686.20 SSRs/Mb). In contrast, *A. alburnus* and *L. rohita* showed the lowest densities (557.45 and 579.01 SSRs/Mb, respectively; Supplementary Table 1). Similar observations have been reported in other teleost genomes, including zebrafish (*Danio rerio*) and rainbow trout (*Oncorhynchus mykiss*), suggesting that SSR abundance is influenced by multiple factors such as ploidy level, lineage-specific repeat expansion, and selective constraints [39,41,42]. This variability reflects the relationship between genome architecture and microsatellite distribution in cyprinid species.

**Figure 3:**
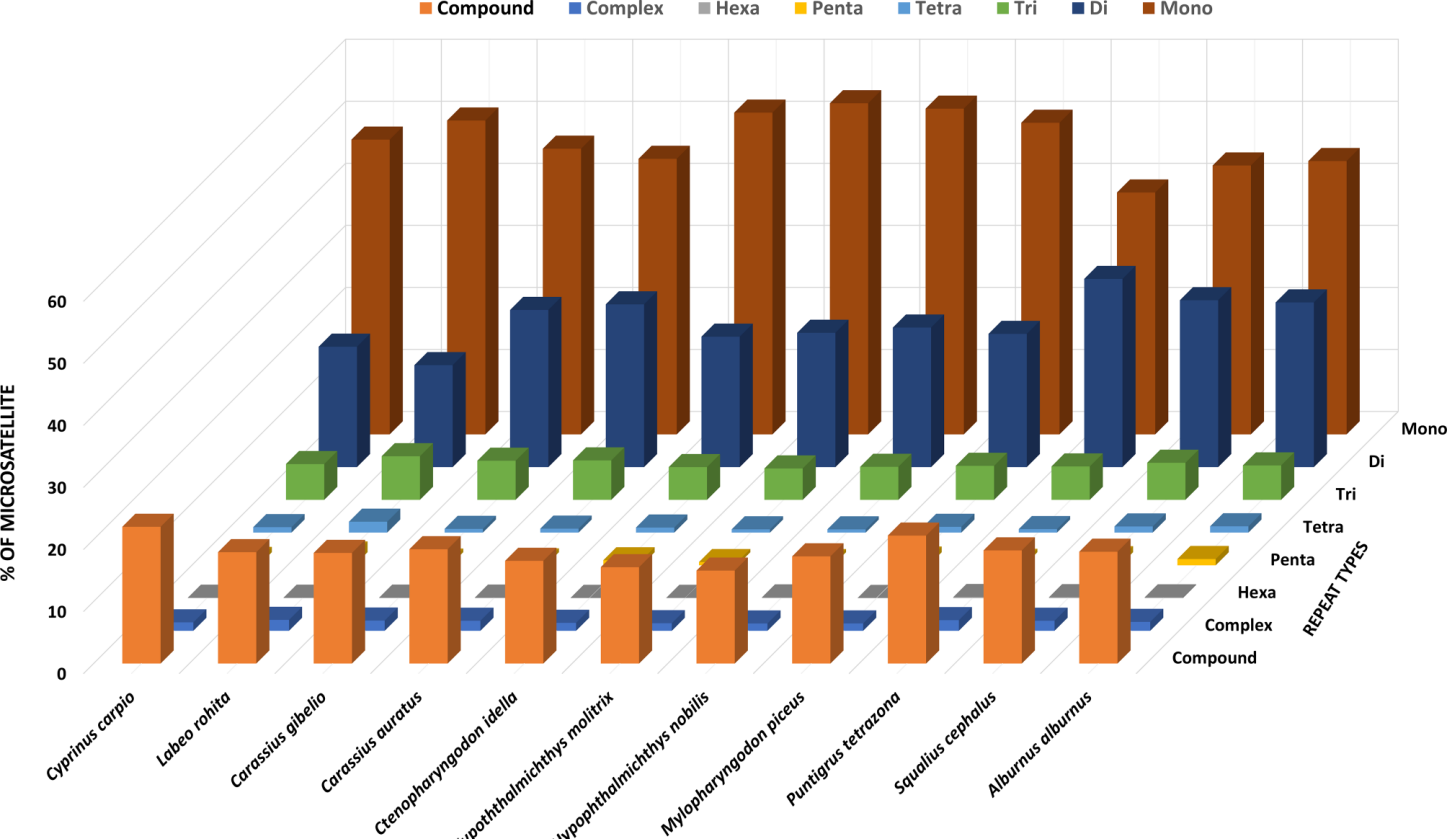
Distribution of microsatellite repeat types across eleven cyprinid species. The y-axis represents the percentage distribution of microsatellite repeat types, while the x-axis lists the species.

Fig. 3 shows the distribution of SSR repeat types across the eleven cyprinid species. Mononucleotide repeats were consistently the most abundant class, followed by dinucleotide repeats, while longer motifs were progressively less frequent. Compound and complex SSRs showed variable representation across species, which may reflect differences in genome structure and evolutionary history. Overall, these patterns suggest that SSR composition is shaped by both intrinsic genomic features and evolutionary processes, contributing to species-specific microsatellite landscapes [43].

### Genomic distribution of SSRs in genic and non-genic regions

**Figure 4:**
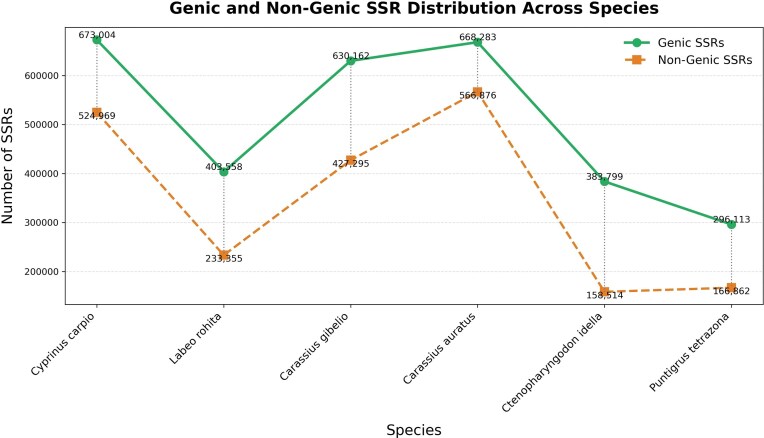
Distribution of genic and non-genic SSRs across six cyprinid species. The y-axis represents the total number of identified SSRs, while the x-axis shows the species.

Analysis of SSR distribution (Fig. 4) revealed a consistent predominance of SSRs within genic regions across all examined cyprinid species. For example, *C. carpio* contained 673,004 genic SSRs compared to 524,969 in non-genic regions, while *C. auratus* showed a similar pattern with 668,283 genic and 566,876 non-genic SSRs. A comparable trend was also observed in species with relatively smaller genomes, such as *P. tetrazona*, where genic SSRs (296,113) exceeded those in non-genic regions (166,862).

SSR distribution was assessed by intersecting SSR coordinates with genome annotation files (GFF/GTF) using BEDTools [34] and comparing their occurrence across genic and non-genic regions. Across species, genic SSRs accounted for approximately 54–71% of total SSRs, indicating a higher representation of SSRs in genic regions compared to non-genic regions (Fig. 4, Supplementary Table 1).

This pattern suggests a non-random distribution influenced by genome organization and functional constraints. SSRs located within genic regions, particularly in coding sequences and untranslated regions, may be subject to selective pressures. For instance, trinucleotide repeats can expand without disrupting the reading frame, which may contribute to their retention in coding regions [44]. In contrast, non-genic regions, although still harboring substantial numbers of SSRs, exhibit comparatively lower abundance, possibly reflecting differences in mutation rates, sequence composition, and selective constraints.

The relatively consistent genic-to-non-genic distribution across species further indicates that these patterns are conserved within cyprinid genomes. Similar trends have been reported in other eukaryotic systems, where SSRs exhibit non-random distribution and are often associated with gene-rich regions, contributing to gene regulation and functional diversification [45,46]. Collectively, these findings indicate that SSR distribution is shaped not only by genome size but also by structural and functional genomic features.

### Repeat motif composition and evolutionary implications

The analysis of individual repeat motifs across the eleven cyprinid species revealed a clear dominance of mononucleotide repeats, particularly A/T-rich motifs. Among these, adenine (A) and thymine (T) repeats were the most abundant across all species, with *C. carpio, C. auratus*, and *C. gibelio* exhibiting the highest counts, consistent with their larger genome sizes. In contrast, guanine (G) and cytosine (C) mononucleotide repeats were comparatively less frequent, a pattern commonly observed in vertebrate genomes, including teleost fishes, due to underlying nucleotide composition biases [45].

Among dinucleotide motifs, AT repeats were predominant across most species, followed by AC and AG motifs, whereas CG repeats were consistently rare, typically occurring at very low frequencies. Notably, AC was the most frequent dinucleotide in *C. carpio, S. cephalus*, and *A. alburnus*, suggesting lineage-specific variation in dinucleotide composition [40]. The depletion of CG motifs is likely attributable to methylation-associated mutational processes in vertebrate genomes, wherein methylated cytosines undergo spontaneous deamination, resulting in C to T transitions and the progressive erosion of CpG dinucleotides over evolutionary time [47].

Trinucleotide repeats exhibited species-specific variation, with AAT and AAC motifs being the most abundant across all eleven species. Other motifs such as ATC, ACT, AGC, and AGG were present at moderate frequencies, whereas GC-rich motifs (e.g. ACG and CCG) were the scarcest, likely reflecting mutational and structural constraints that limit the expansion of GC-rich repeats. The predominance of A/T-rich motifs is consistent with well-established mutational biases favoring AT enrichment and the relative instability of GC-rich sequences in vertebrate genomes [47].

Collectively, these findings highlight the influence of mutational bias, DNA methylation dynamics, and lineage-specific evolutionary processes in shaping the microsatellite landscape of cyprinid genomes. Such patterns reflect both intrinsic genomic properties and selective constraints acting on repeat stability and expansion (Supplementary Table 2). Importantly, this motif-level characterization has direct practical implications, as the enrichment of A/T-rich di- and trinucleotide repeats, particularly within genic regions, provides a valuable resource for developing highly polymorphic SSR markers for population genetics, conservation studies, and marker-assisted selection in aquaculture species.

Beyond their compositional patterns, SSRs play a crucial role in shaping genomic and phenotypic diversity. Their high mutation rates, primarily driven by replication slippage, generate substantial length polymorphism within and between species [43]. This variability contributes to genetic diversity, which is essential for adaptation to changing environments and evolutionary processes [48]. SSRs located within coding regions can influence protein structure by altering amino acid repeat lengths, whereas those in regulatory regions may modulate gene expression by affecting transcription factor binding or chromatin accessibility [45,49]. Such functional impacts can lead to phenotypic variation, influencing traits related to growth, stress response, and environmental adaptability.

The high polymorphic nature of SSRs further underpins their utility as molecular markers in genetic studies. Their ability to distinguish closely related genotypes makes them particularly valuable for linkage mapping, population structure analysis, and biodiversity assessment. Despite the increasing availability of SNP-based markers, SSRs remain widely used due to their co-dominant inheritance, reproducibility, and relatively simple laboratory requirements. In the context of cyprinid species, the identified SSR repertoire provides a robust foundation for marker-assisted breeding, stock management, and conservation strategies [50].

Together, these observations emphasize the dual role of microsatellites as both functional genomic elements and powerful molecular tools. Their distribution and variability not only reflect underlying evolutionary processes but also provide critical resources for advancing genetic research and aquaculture improvement programs.

### Database architecture and utilities

**Figure 5:**
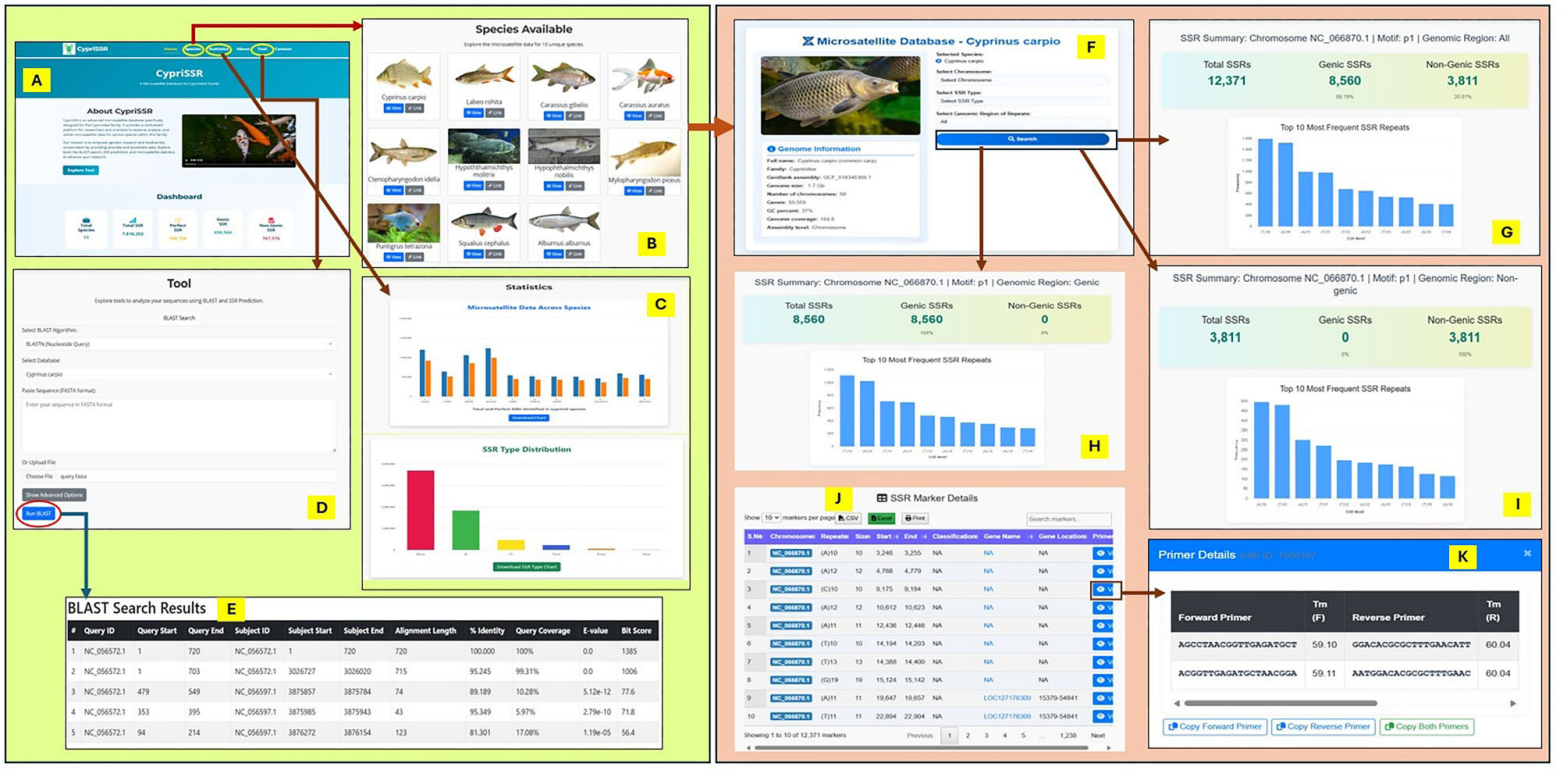
Overview of the *CypriSSR* database workflow and user interface.(A) Homepage providing access to database tools and general information. (B) Species selection panel displaying available fish species.(C) Statistics page showing SSR distribution and summary statistics.(D) Tool section for sequence input and BLAST search execution. (E) Example BLAST results displaying alignment metrics. (F) Species-specific SSR database view with detailed genomic information.(G) SSR summary across genomic regions showing total, genic, and non-genic SSRs. (H) SSR summary for genic regions. (I) SSR summary for non-genic regions. (J) SSR marker details Table with locus-specific information. (K) Primer details including sequence and properties.

The *CypriSSR* interface comprises six major tabs: Home, Species, Statistics, Tool, About, and Contact, designed to provide a structured and user-friendly environment for accessing genome-wide SSR data in cyprinid fishes. The ‘Home’ tab serves as the entry point to the database, providing an overview of *CypriSSR* along with a concise introduction to SSRs and their significance in genomics and molecular breeding. It also features an integrated dashboard that summarizes key dataset statistics, including the total number of species, total SSRs, perfect SSRs, and their distribution across genic and non-genic regions. This overview enables users to rapidly assess the scale, composition, and biological relevance of the dataset prior to detailed exploration. An overview of the CypriSSR database interface, including key navigation features and design elements, is shown in Fig. 5.

The ‘Species’ tab functions as the core data exploration component, offering a visually organized catalog of cyprinid species, each represented with images and quick-access options to enhance navigation. Upon selection of a species, users are directed to a dedicated species-specific interface that seamlessly integrates genomic metadata with SSR querying capabilities. The Genome Information panel provides essential assembly-level details, including species name, family, genome assembly accession, genome size, chromosome number, gene count, GC content, genome coverage, and assembly level. These metadata provide critical context for interpreting SSR abundance and distribution patterns. The query interface enables users to refine SSR searches using multiple parameters, such as chromosome selection, SSR motif type, and genomic location (genic or non-genic regions). The resulting SSR loci are displayed in an interactive and searchable tabular format containing detailed annotations, including repeat motif, repeat count, genomic coordinates, and associated primer information. The Table supports dynamic filtering and sorting, along with export functionalities that allow users to download results in standard formats such as CSV and Excel for downstream computational or experimental analyses.

The ‘Statistics’ tab provides comprehensive graphical summaries of SSR distribution and composition across species. It includes comparative visualizations of total and perfect SSR counts among different cyprinid species, as well as distribution profiles of SSR motif classes, including mono-, di-, tri-, tetra-, penta-, and hexanucleotide repeats. These visual analytics enable users to identify trends in microsatellite abundance, motif composition, and genome-wide distribution patterns, thereby facilitating comparative genomics and evolutionary studies. The tab also supports downloading of high-resolution charts, allowing users to incorporate statistical outputs into publications, reports, and presentations.

The ‘Tool’ tab incorporates a BLAST-based sequence analysis interface, enabling users to perform similarity searches against the *CypriSSR* database. It supports both *blastn* and *tblastn* algorithms, providing flexibility for nucleotide- and protein-based queries. Users can input sequences directly in FASTA format or upload sequence files for batch processing. The interface includes advanced parameter settings, allowing customization of search criteria such as alignment thresholds and scoring parameters. Upon execution, the system performs sequence alignment and presents the results in a structured tabular format, including alignment scores, sequence identity, E-values, and positional information. Interactive links enable users to navigate directly to corresponding SSR entries, facilitating the identification of homologous SSR regions and assessment of marker transferability across species.

Hyperlinked genomic coordinates further enhance the usability of *CypriSSR* by allowing users to explore SSR loci in relation to gene annotations and other genomic features. This integration supports functional interpretation of SSRs, including their potential roles in gene regulation, environmental adaptation, and trait-associated variation. Notably, analysis of SSR distribution across representative cyprinid species revealed that genic SSRs constitute approximately 59.7% of total SSRs, suggesting their potential involvement in functional and regulatory processes. In contrast, SSRs located in non-genic regions may contribute to genome regulation through mechanisms such as chromatin remodeling, DNA methylation, and modulation of gene expression dynamics [51]. These findings underscore the broader biological significance of SSRs beyond their traditional role as molecular markers.

The ‘About’ and ‘Contact’ tabs provide detailed information regarding database development, data sources, and implementation, while also offering users a platform to communicate with the developers for queries, feedback, and future updates. The database is designed with scalability in mind, allowing incorporation of additional species, updated genome assemblies, and enhanced analytical features in future versions.

**Figure 6:**
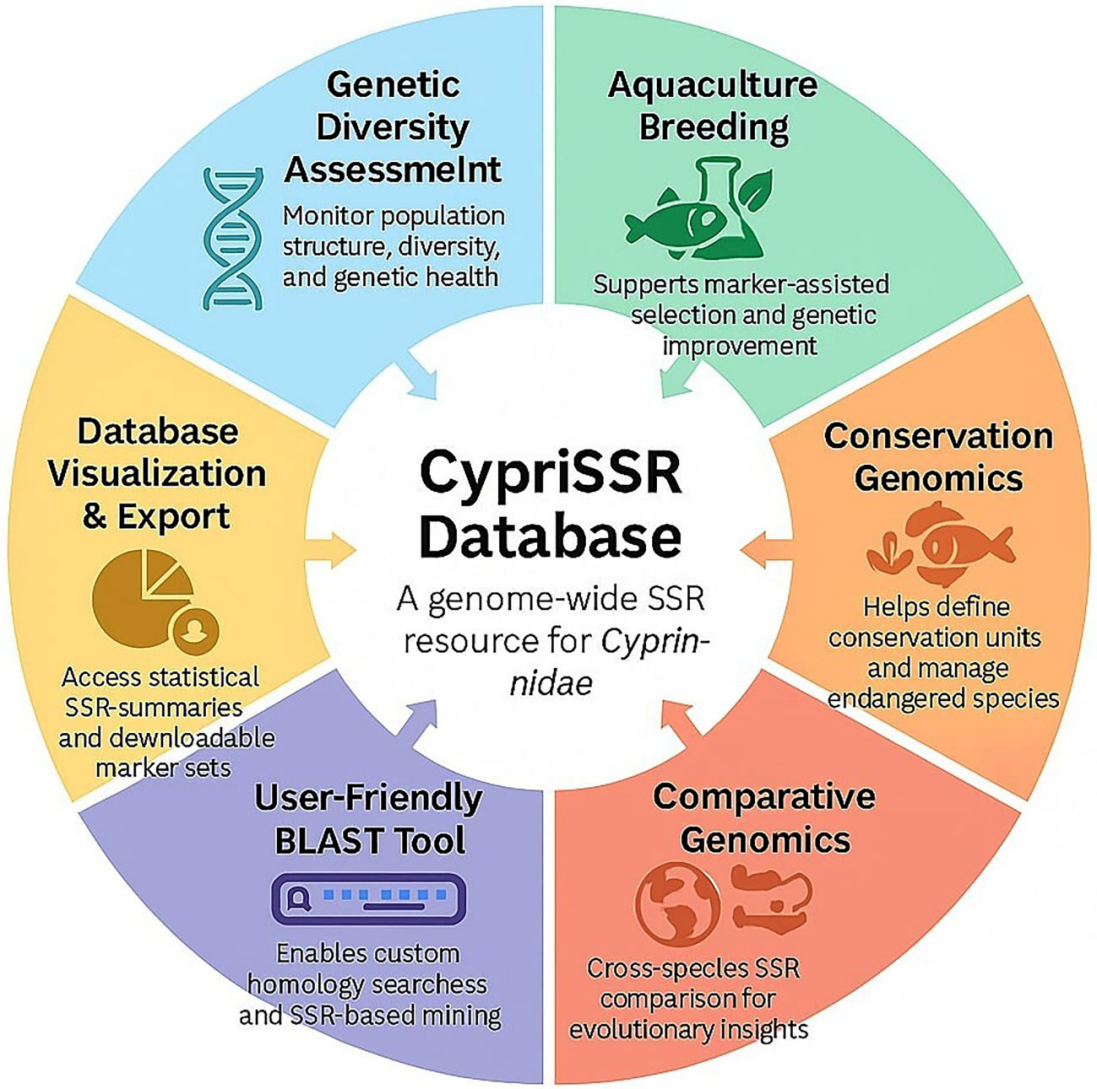
Key functionalities and applications of the *CypriSSR* database. This graphical summary highlights major features, including genetic diversity assessment, aquaculture breeding, conservation genomics, comparative genomics, a user-friendly BLAST tool, and database visualization and export capabilities.

*CypriSSR* is an interactive web-based resource developed for cyprinid species that provides a user-friendly environment for exploring genome-wide SSR data. The platform enables users to perform queries using multiple parameters, including chromosome, repeat type, motif class, and genomic location, allowing flexible and precise data retrieval. The output is displayed in a structured tabular format containing detailed annotations such as motif sequence, repeat count, genomic coordinates, and primer information, with options to download results in formats such as CSV and Excel. Integrated links associated with SSR entries allow users to access related genomic context and annotations, improving the interpretability and utility of identified markers. The database also includes comprehensive statistical summaries of SSR distribution across species and repeat types, facilitating comparative analyses. Designed with simplicity and functionality in mind, the interface supports smooth navigation across pages, ensuring ease of use for researchers. *CypriSSR* is structured to accommodate future updates, including the addition of new species, improved genome assemblies, and enhanced annotations, thereby maintaining its relevance as a dynamic resource for cyprinid genomics and aquaculture research. The breadth of CypriSSR’s applications is illustrated in Fig. 6, which highlights six core functional domains: genetic diversity assessment, aquaculture breeding, conservation genomics, comparative genomics, homology-based sequence searches via the BLAST interface, and integrated visualization and data export tools. As shown, CypriSSR enables users to assess population structure, develop markers for selective breeding, delineate conservation units, perform cross-species comparisons of SSR profiles, conduct custom sequence similarity searches, and retrieve summary statistics or tailored marker sets. Collectively, these features establish CypriSSR as a comprehensive and versatile resource for advancing cyprinid genetics, conservation biology, and sustainable aquaculture research.

*CypriSSR* is freely accessible online at: http://46.202.167.198/fishssr

## Conclusion

*CypriSSR*, a comprehensive genome-wide SSR database developed for cyprinid fishes, integrates over 7.8 million SSR loci identified from 11 chromosome-level genome assemblies. This resource provides detailed annotations, including repeat motifs, genomic locations, genic classifications, and primer information, enabling efficient marker discovery and utilization. The observed predominance of A/T-rich mononucleotide repeats, along with the enrichment of di- and trinucleotide motifs in genic regions, highlights their potential functional roles in gene regulation and adaptive evolution. Furthermore, the substantial variation in SSR abundance and density across species reflects the dynamic nature of cyprinid genomes and their evolutionary complexity. By combining large-scale genomic data with an interactive and accessible interface, *CypriSSR* enables flexible querying, cross-species comparison, and sequence-based exploration through integrated tools such as BLAST. This platform provides a valuable foundation for applications including genetic diversity analysis, population structure studies, molecular breeding, and conservation genomics. The availability of genome-wide SSR markers, particularly those located in functionally relevant regions, offers significant potential for marker-assisted selection and germplasm improvement in aquaculture species.

*CypriSSR* can be further expanded by incorporating additional cyprinid species and updated genome assemblies as they become available. Continued improvements in database functionality and the inclusion of analytical tools for marker validation and cross-species applications will further enhance its utility for cyprinid genomics and breeding research.

## Supplementary Material

baag035_Supplemental_Files
